# Pathways to new drug discovery in neuropsychiatry

**DOI:** 10.1186/1741-7015-10-151

**Published:** 2012-11-29

**Authors:** Michael Berk

**Affiliations:** 1 Deakin University, School of Medicine, Barwon Health, P.O. Box 291, Geelong, 3220, Australia; 2 Orygen Youth Health Research Centre, 35 Poplar Rd, Parkville, 3052, Australia; 3 Centre of Youth Mental Health, University of Melbourne, 35 Poplar Rd, Parkville, 3052, Australia; 4 Florey Institute for Neuroscience and Mental Health, University of Melbourne, Kenneth Myer Building, 30 Royal Parade, 3052, Parkville, Australia; 5 Department of Psychiatry, University of Melbourne, Level 1 North, Main Block, Royal Melbourne Hospital, Parkville, 3052, Australia

**Keywords:** depression, bipolar disorder, diabetes, treatment, drug discovery, pathophysiology

## Abstract

There is currently a crisis in drug discovery for neuropsychiatric disorders, with a profound, yet unexpected drought in new drug development across the spectrum. In this commentary, the sources of this dilemma and potential avenues to redress the issue are explored. These include a critical review of diagnostic issues and of selection of participants for clinical trials, and the mechanisms for identifying new drugs and new drug targets. Historically, the vast majority of agents have been discovered serendipitously or have been modifications of existing agents. Serendipitous discoveries, based on astute clinical observation or data mining, remain a valid option, as is illustrated by the suggestion in the paper by Wahlqvist and colleagues that treatment with sulfonylurea and metformin reduces the risk of affective disorder. However, the identification of agents targeting disorder-related biomarkers is currently proving particularly fruitful. There is considerable hope for genetics as a purist, pathophysiologically valid pathway to drug discovery; however, it is unclear whether the science is ready to meet this promise. Fruitful paradigms will require a break from the orthodoxy, and creativity and risk may well be the fingerprints of success.

See related article http://www.biomedcentral.com/1741-7015/10/150

## Introduction

Before applying a microscope to a problem, it is always wise to first use a wide-angle lens. In psychiatric disorders, as in most of medicine, the two things that matter most to people with disorders are the availability of effective treatments for those disorders and the capacity to prevent these disorders occurring in the first instance. With regard to the latter, psychiatry is only beginning to develop an evidence base of potentially plastic risk factors for the development of common mental disorders. Existing treatments for most major psychiatric disorders are limited in terms of their efficacy and tolerability. The situation in terms of new drug development is best summarized by Steven Hyman, a previous director of the US National Institute of Mental Health (NIMH) director, who stated that, 'Drug discovery is at a near standstill for treating psychiatric disorders such as schizophrenia, bipolar disorder, depression, and common forms of autism. Despite high prevalence and unmet medical need, major pharmaceutical companies are deemphasizing or exiting psychiatry, thus removing significant capacity from efforts to discover new medicines' [1].

## Pathways to drug discovery

How did we get into this pickle? And how can we extricate ourselves? To answer this, it is necessary we look at the pathways to drug discovery and critically analyze which of these are fruitful paths to drug discovery and what new avenues might be open. The initial path has been that of serendipity. Almost all psychotropic agents have been discovered accidentally, their mechanisms of action reverse-engineered, and new agents developed that, in some cases, have improved on the originals. Lithium, the antipsychotics, and the antidepressants all fall into this class. Indeed, most of our knowledge of the neurobiology of neuropsychiatric disorders derives from this process; this includes the dopamine theory of schizophrenia and the monoamine theory of antidepressants [2]. Sadly, the rewards deriving from this path seem to be dwindling. At least part of the problem arises from the fact that these agents have been utilized for proof of concept in animal models of these disorders, and these models in a circular manner seem to reflect the profile of the original agents and are of dubious value in detecting novel mechanisms [3].

Nevertheless, both astute clinical examination, and observational studies of unexpected benefits of agents used for other indications, remain valuable avenues for drug discovery, despite their lack of a primary, pathophysiologically grounded scientific process. In this context, the paper by Wahlqvist *et al. *[4], which used a retrospective case-control study of data from the Taiwanese National Health Insurance database to examine whether people taking a sulfonylurea and/or metformin had a lower incidence of depression, is noteworthy. The authors reported that patients with diabetes taking sulfonylurea or metformin had a lower incidence of depression than people not taking these anti-diabetic agents [4]. This is not entirely unexpected. There are published data suggesting that pioglitazone, an oral anti-diabetic agent, also has antidepressant properties [5]. The hypothesis leading the authors of this study on pioglitazone was that this agent has anti-inflammatory properties, which remains a plausible explanation for its efficacy profile [5]. Nevertheless, it is entirely plausible that this group of agents may be operating through entirely different pathways, which substantially increases the gravity of the finding. Shared operative pathways between depression and diabetes have been indicated by a number of studies that suggest that depression and diabetes are risk factors for each other [6,7]. It is noteworthy that oral hypoglycemic agents might have protective effects against other neuropsychiatric disorders, including dementia and Parkinson's disease [8,9], suggesting that these agents could have effects on pathways to neuroprogression that may be overlapping in these disorders.

Another promising pathway of new drug discovery for neuropsychiatric disorders is to target biomarkers known to be dysregulated in these disorders. The most promising of these seems to be the inflammatory and oxidative pathways. As exemplars of this approach, N-acetyl cysteine, a precursor of glutathione, the brain's principal redox scavenger, has been shown to be efficacious for a diverse range of neuropsychiatric disorders, including schizophrenia, bipolar disorder, autism, and addictions [10,11]. Exploring the use of agents with anti-inflammatory properties has also been fruitful, with agents as diverse as statins, aspirin, celecoxib, and pioglitazone showing promise [12,13]. It can be argued that this is, at the present time, the most productive route.

The revolution in genetics has opened an entirely new vista for new drug discoveries. Indeed, many of the most influential funding agencies such as the National Institutes of Health (NIH) in the USA are prioritizing this avenue of research. The promise of genetics is considerable, because of the lack of a coherent pathophysiology for almost all psychiatric disorders and hence rational drug targets for such disorders. Although psychiatric genetics remains in its infancy, a number of promising candidates have emerged, including the calcium channel proteins CACNA1 (Calcium channel, voltage-dependent, T type, alpha 1) and IKCNH2 (potassium voltage-gated channel, subfamily H (eag-related), member 2), vasoactive intestinal peptide 2, DISC1 (Disrupted in schizophrenia 1) and Ankyrin-3 [14]. Nevertheless, several caveats are necessary. Very few of the genes of interest involve traditional neurotransmitter targets, the source of most current psychotropics. Hundreds of genes are involved, all with very small effects, and their physiology and pathophysiology remains unknown. I am constantly reminded of the day, as a young neurology registrar, that the professor walked in, enthused with excitement, to tell us that the gene for Huntington's disease (HD) had been discovered. The experts were sure, given that there is a single gene coding for a single protein and accounting for 100% of the disease expression, that a treatment for HD would be found within a year or two. It is many decades since that day, and no treatment has been forthcoming. As psychiatric genetics is vastly more complicated, hopes have to be cautious at best. Although genetics offers an avenue of scientific purity, it is likely that considerable time will need to pass before the technology is able to utilize the un-translated genetic data to develop new psychiatric treatments.

In the context of drug discovery, a word about diagnosis is appropriate. Current diagnostic classifications are entirely based on phenomenology. In no other branch of medicine does phenomenology track pathology. There are multiple causes of every symptom and diseases that can be expressed with a dizzying array of seemingly unrelated phenomenology. Highlighting the limitations of our current classification, biomarkers including cognition, neuroimaging, inflammation, oxidative stress, and neurotropins show absolutely no respect for the current classifications, nor might we expect that they should [15,16]. In this regard, the use of traditional diagnostic categories for drug discovery is more of a blinker than a lens, and approaches that eschew current classifications need to be explored.

There is a bizarre paradox that hard-won improvements in service delivery, diagnosis, and access to treatments might be hampering the ability to discover novel therapies. For example, in antidepressant clinical studies, drug placebo differences have been shrinking by about two points per year over the past four or five decades. Indeed, it is becoming increasingly difficult to demonstrate the efficacy of even established antidepressants. This is highly unlikely to be entirely due to our inability to discover useful compounds; it is much more likely to be powerfully influenced by an artifact of the process used in conduct of clinical trials. Fifty years ago, when the first studies commenced, severely ill people, who had not previously been exposed to treatments, were the primary study population. Hence, only small studies were required to show large clinical effects. Two parallel processes have occurred since then. First, diagnostic categories are being used for increasingly mildly ill populations, leading to a growth in perceived prevalence, but a dilution of 'endogenous' or biologic factors. Second, service filters have a powerful effect on study populations. Clear clinical-care pathways exist for depressed individuals, for example, who are likely to seek treatment in primary-care settings, which have been substantially upskilled of late. Treatment-responsive individuals benefitting from primary or secondary care are consequently unavailable for clinical trials. Thus, clinical-trial populations tend to select individuals who have failed on or are disillusioned with existing therapeutic options [17]. It is unlikely that our capacity to develop novel therapies is going to increase unless we are able to address these fundamental issues.

## Future direction and conclusions

The receding tide of infective disorders, trauma, and many major medical disorders, resulting from improved general medical preventative, diagnostic and treatment strategies, leaves psychiatric disorders exposed as the predominant burden of disability in the developed world. The imperative to discover new treatments is clear, not only because of the aforementioned issues, but because of the limitations of established treatments. It is clear that we need to move beyond high-throughput screening of compounds aimed at historical targets; we need to explore fundamentally different pathways to drug discovery such as genetics. However, as noted, this is a high-risk, very low-probability, albeit high-return scenario. Increasing attention to biomarker-indicated targets is likely to be a very promising avenue, but we must not lose sight of the fact that serendipity and keen clinical and scientific observation remains the most fruitful pathway for the discovery of novel therapies.

## Competing interests

MB has received Grant/Research Support from the NIH, Cooperative Research Centre, Simons Autism Foundation, Cancer Council of Victoria, Stanley Medical Research Foundation, MBF, NHMRC, Beyond Blue, Rotary Health, Geelong Medical Research Foundation, Bristol-Myers Squibb, Eli Lilly, GlaxoSmithKline, Meat and Livestock Board, Organon, Novartis, Mayne Pharma, Servier, and Woolworths; has been a speaker for Astra Zeneca, Bristol-Myers Squibb, Eli Lilly, GlaxoSmithKline, Janssen Cilag, Lundbeck, Merck, Pfizer, Sanofi Synthelabo, Servier, and Solvay and Wyeth; and has served as a consultant to AstraZeneca, Bristol-Myers Squibb, Eli Lilly, GlaxoSmithKline, Janssen Cilag, Lundbeck Merck, and Servier.

## Author's Information

MB is currently the Alfred Deakin Professor of Psychiatry in the School of Medicine, Deakin University, and is also a Professorial Research fellow at the University of Melbourne in the Department of Psychiatry, the Centre for Youth Mental Health, Orygen Research Centre, and the Florey Institute for Neuroscience and Mental Health. His predominant interests include risk factors for and prevention of mood disorders, and the discovery and implementation of novel therapies.

## Pre-publication history

The pre-publication history for this paper can be accessed here:

http://www.biomedcentral.com/1741-7015/10/151/prepub
